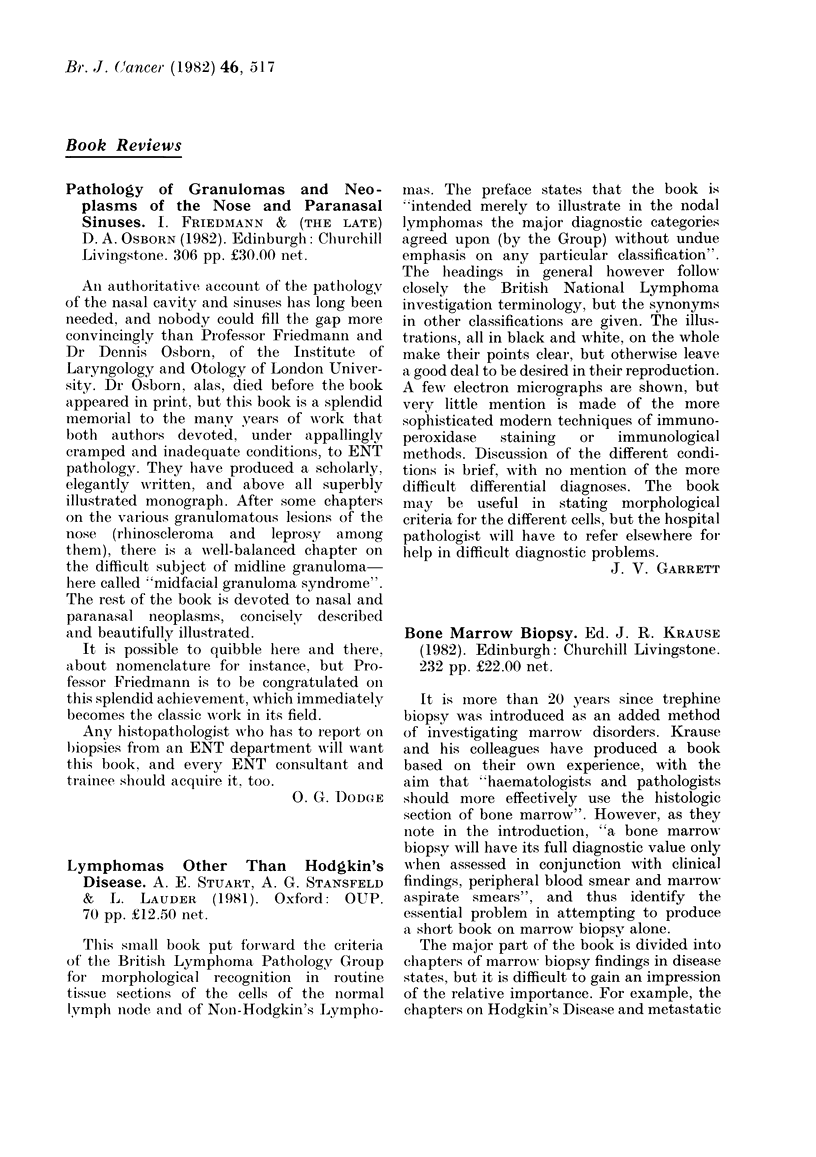# Lymphomas Other Than Hodgkin's Disease

**Published:** 1982-09

**Authors:** J. V. Garrett


**Lymphomas Other Than Hodgkin's Disease.** A. E. Stuart, A. G. Stansfeld & L. Lauder (1981). Oxford: OUP. 70 pp. £12.50 net.

This small book put forward the criteria of the British Lymphoma Pathology Group for morphological recognition in routine tissue sections of the cells of the normal lymph node and of Non-Hodgkin's Lymphomas. The preface states that the book is "intended merely to illustrate in the nodal lymphomas the major diagnostic categories agreed upon (by the Group) without undue emphasis on any particular classification". The headings in general however follow closely the British National Lymphoma investigation terminology, but the synonyms in other classifications are given. The illustrations, all in black and white, on the whole make their points clear, but otherwise leave a good deal to be desired in their reproduction. A few electron micrographs are shown, but very little mention is made of the more sophisticated modern techniques of immunoperoxidase staining or immunological methods. Discussion of the different conditions is brief, with no mention of the more difficult differential diagnoses. The book may be useful in stating morphological criteria for the different cells, but the hospital pathologist will have to refer elsewhere for help in difficult diagnostic problems.

J. V. Garrett